# COVID-19-associated acute cerebral venous thrombosis: clinical, CT, MRI and EEG features

**DOI:** 10.1186/s13054-020-03131-x

**Published:** 2020-07-11

**Authors:** Fabian Roy-Gash, De Mesmay Marine, Devys Jean-Michel, Vespignani Herve, Blanc Raphael, Engrand Nicolas

**Affiliations:** 1 Neuro-Intensive Care Unit, Fondation Ophtalmologique Adolphe de Rothschild, 29 rue Manin, 75019 Paris, France; 2 Serenity Medical Services—NeuroPhy, ICM-iPEPS, 47-85 Boulevard de l’Hôpital, 75013 Paris, France; 3 Department of Interventional Neuro-Radiology, Fondation Rothschild, 29 rue Manin, 75019 Paris, France

**Keywords:** Cerebral venous thrombosis, Intracerebral hematoma, Electroencephalography, SARS-CoV-2, COVID-19, Pandemics, Diagnostic imaging

Dear editor,

Many recent COVID-19 series have reported arterial or venous thrombosis (stroke, pulmonary embolism, etc.) [1, 2]. Here, we report a case of COVID-19 associated cerebral venous thrombosis (CVT) with dramatic evolution.

On April 3, 2020, a 63-year-old female presented to the emergency department because of aphasia and right hemiplegia. She had a 12-day history of fever, cough, and anosmia. Her husband was hospitalized in intensive care for confirmed COVID-19 acute respiratory distress syndrome (ARDS). Brain MRI showed a large left temporal brain hemorrhage and a suspicion of CVT confirmed on a venous brain CT scan and chest CT showed typical COVID-19 patchy ground-glass opacities in both lungs (Fig. 1).

**Fig. 1:**
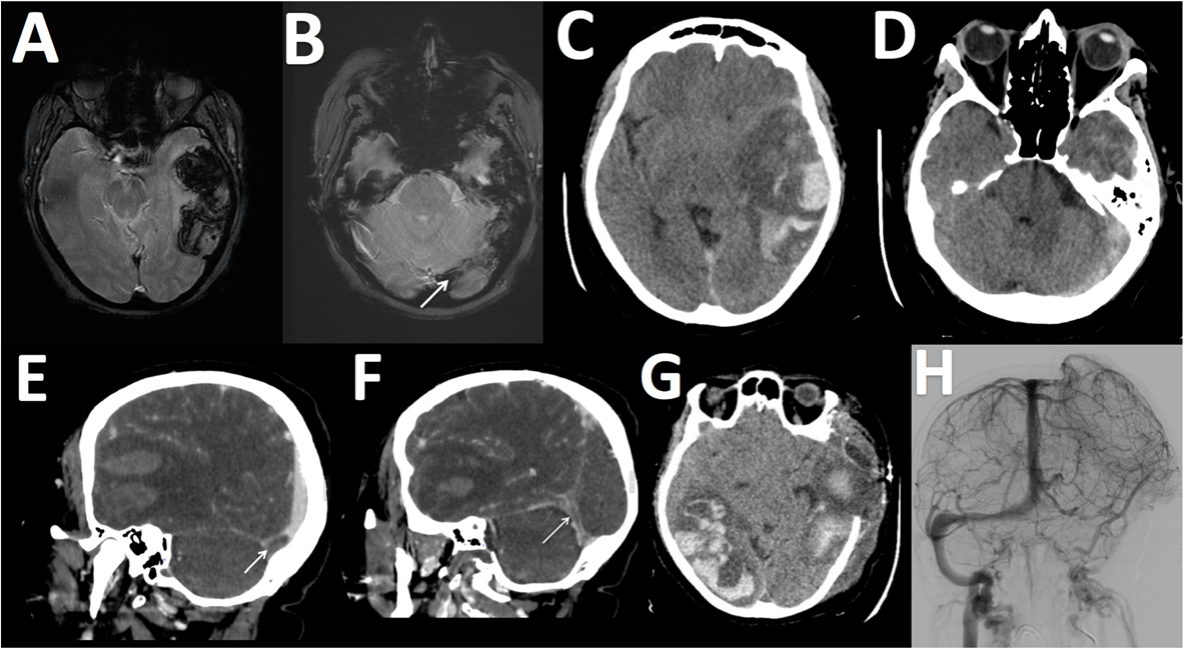
MRI, venous CT scanner, and cerebral angiography at admission and day 14. MRI images (**a**, **b**) demonstrates voluminous left temporal hemorrhage with venous thrombosis (arrow). Venous CT scanner (**c**–**f**) confirms the existence of the extensive venous thrombosis. Located in the straight sinus and left lateral sinus (arrow). Day 14 CT scanner shows contro-lateral. Brain hemorrhage (**g**) and cerebral angiography shows persistent left thrombosis (**h**)

The patient suddenly suffered a clinical status epilepticus and was administered i.v. lacosamide.

Laboratory results showed hyperfibrinogenemia (7.2 g/L) and high ferritin levels (1427 μg/L).

The nasopharyngeal and bronchial samples were negative for SARS-CoV-2.

Most common causes of genetic thrombotic disorders and antiphospholipid antibody syndrome were excluded.

The patient was started on an intravenous curative dose of heparin anticoagulation.

Electroencephalograpy (EEG) showed background theta activity unreactive to nociceptive stimulus, with pseudo-periodic activity of a short period composed of slow di-phasic waves irradiating towards the anterior regions (Fig. 2). Although subtle status epilepticus could not be excluded, the aspect was not typical and other successive EEG traces would confirm this non-epileptic paroxystic pseudo-periodic pattern. The patient eventually underwent surgical intracranial hematoma evacuation followed by decompressive craniectomy.

**Fig. 2:**
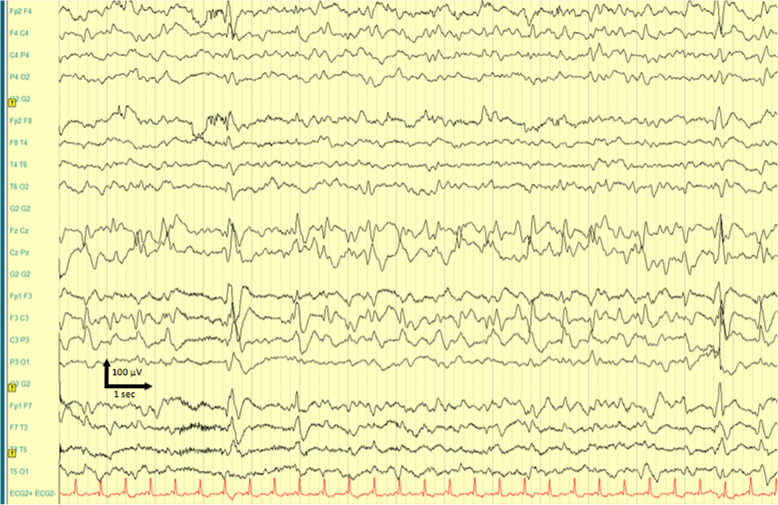
EEG findings on day 2 in ICU revealing background asymetric slow left frontotemporal theta activity, unreactive to nociceptive stimulus, with pseudo-periodic activity of short period composed of slow di-phasic waves irradiating towards the anterior contro-lateral regions. Scale 15 s, 100 μV/mm, longitudinal

On April 17th, brain CT scan revealed a new intracranial contralateral bleeding most likely following contralateral venous thrombosis despite being properly treated with intravenous heparin. Venous angiography showed persistent left thrombosis (Fig. 1). On April 25th, the patient was tested positive for SARS-CoV-2 plasmatic IgG and IgM (ELISA test).

On April 29th, the patient died following therapeutic limitation after ethical consultation group expertise.

Although both samples were negative for SARS-CoV-2, we considered the patient infected by it, given the initial symptomatology, the confirmed infection in one relative, the specific aspect of the thoracic CT scan [3], and the positive serology. Furthermore, in this case, the thrombotic event occurred 12 days after the first influenza-like symptoms, which corresponds to the most inflammatory period of COVID-19 [4, 5].

In addition to the left lesion temporal focus observed on the EEG, the background activity and paroxysmal activity describes atypical patterns, which can be mistaken with persistent epileptic activity. However, we believe it is compatible with newly described patterns of specific COVID-19 encephalopathy [6].

Overall, this case suggests that practitioners should be aware of the possibility of a CVT in this novel COVID-19 context, especially during the post-viral period.

## Data Availability

All data analyzed in this report is available by simple request to the corresponding author.

## Abbreviations

ARDS: Acute respiratory distress syndrome
COVID-19: Coronavirus disease 19
CVT: Cerebral venous thrombosis
EEG: Electroencephalography

## References

[1] Shi S, Qin M, Shen B, et al: Association of cardiac injury with mortality in hospitalized patients with COVID-19 in Wuhan, China. JAMA Cardiol. 2020;doi:10.1001/jamacardio.2020.0950. 10.1001/jamacardio.2020.0950 PMC7097841 32211816

[2] Mao L, Jin H, Wang M, et al. Neurologic manifestations of hospitalized patients with coronavirus disease 2019 in Wuhan, China. JAMA Neurol. 2020. 10.1001/jamaneurol.2020.1127. 10.1001/jamaneurol.2020.1127 PMC7149362 32275288

[3] Fang Y, Zhang H, Xie J, et al: Sensitivity of chest CT for COVID-19: comparison to RT-PCR. Radiol. 2020;doi:10.1148/radiol.2020200432. 10.1148/radiol.2020200432 PMC7233365 32073353

[4] Siddiqi HK, Mehra MR: COVID-19 illness in native and immunosuppressed states: a clinical-therapeutic staging proposal. J Heart Lung Transplant. 2020;doi:10.1016/j.healun.2020.03.012. 10.1016/j.healun.2020.03.012 PMC7118652 32362390

[5] Tang N, Li D, Wang X, Sun Z (2020). Abnormal coagulation parameters are associated with poor prognosis in patients with novel coronavirus pneumonia. J Thromb Haemost.

[6] Vespignani H, Colas D, Lavin BS, et al. Report of EEG Finding on Critically Ill Patients with COVID-19 [published online ahead of print, 2020 Jun 13]. Ann Neurol. 2020; 10.1002/ana.25814. 10.1002/ana.25814 PMC7323170 32533727

