# Correction to: COVID-19-associated acute cerebral venous thrombosis: clinical, CT, MRI and EEG features

**DOI:** 10.1186/s13054-020-03225-6

**Published:** 2020-08-28

**Authors:** Fabian Roy-Gash, Marine De Mesmay, Jean-Michel Devys, Hervé Vespignani, Raphaël Blanc, Nicolas Engrand

**Affiliations:** 1 Neuro-Intensive Care Unit, Fondation Ophtalmologique Adolphe de Rothschild, 29 rue Manin, 75019 Paris, France; 2 Serenity Medical Services—NeuroPhy, ICM-iPEPS, 47-85 Boulevard de l’Hôpital, 75013 Paris, France; 3 Department of Interventional Neuro-Radiology, Fondation Rothschild, 29 rue Manin, 75019 Paris, France

**Correction to: Crit Care (2020) 24: 419**

**https://doi.org/10.1186/s13054-020-03131-x**

Following publication of the original article [1], the authors identified an error in five author names. The given name and family name were erroneously transposed.

The incorrect author names are:

De Mesmay Marine

Devys Jean-Michel

Vespignani Herve

Blanc Raphael

Engrand Nicolas

The correct author names are:

Marine De Mesmay

Jean-Michel Devys

Hervé Vespignani

Raphaël Blanc

Nicolas Engrand

The author group has been updated above and the original article [1] has been corrected.
